# Aneurysmal Bone Cyst Plus Lesions: A Case Report and a Literature Review

**DOI:** 10.7759/cureus.27912

**Published:** 2022-08-12

**Authors:** Archana Sonone, Alka Hande, Madhuri N Gawande, Swati K Patil, Aayushi Pakhale

**Affiliations:** 1 Department of Oral Pathology and Microbiology, Sharad Pawar Dental College and Hospital, Datta Meghe Institute of Medical Sciences (Deemed to be University), Wardha, IND

**Keywords:** unicystic lesion, who classification, ossifying fibroma, aneurysmal bone cyst, odontogenic cyst

## Abstract

The intraosseous osteolytic lesions mainly involving the metaphyseal region of vertebrae and long bones were diagnosed as aneurysmal bone cysts (ABCs). Further, an ABC was known as an ossifying hematoma. It is considered an expanding osteolytic lesion consisting of blood-filled spaces of variable sizes separated by connective tissue septa containing trabeculae of osteoid tissue and osteoclast giant cells. It is frequently reported to involve long bones; however, only 1.9% prevalence is seen in jaw bones. It represents a very small percentage of all non-odontogenic tumors. ABC shows variations in age prevalence and its clinical presentation may be challenging to the surgeon. In addition, ABC may occur in association with other primary bone pathologies like ossifying fibroma, fibrous dysplasia, and giant cell tumor; such entities are known as ABC plus lesions. Here we present a classic case of ABC plus lesion.

## Introduction

An aneurysmal bone cyst (ABC) was first documented as an ossifying hematoma by Van Arsadel in 1893. Jaffe and Lichestine named ABC to intraosseous, osteolytic lesions mainly involving the metaphyseal region of vertebrae and long bones [1]. Bernier and Bhaskar reported the first case of ABC in 1958. In 2005, WHO categorized this lesion as an expanding osteolytic lesion consisting of blood-filled spaces of variable sizes separated by connective tissue septa containing trabeculae of osteoid tissue and osteoclast giant cells [2]. 

It is most frequently detected in long bones (50%) and vertebral columns (20%); in the jaw bones, only 1.9% incidence is present, representing 1.5% of all non-odontogenic tumors. The mandible is more commonly affected than the maxilla (3:1). In the mandible, the body of the mandible, ramus, and angle of the mandible are predominantly involved. Younger people are affected more frequently than elderly people [3]. ABC has two clinicopathological variants, one is the primary ABC and the other is the secondary ABC which is derived from the secondary lesion. Primary ABC lesion is again further divided into congenital and acquired forms. Congenital ABC is established from arteriovenous malformation, growth, and maturation of teeth in infancy. The acquired subtype of primary ABC can be explained by its association with trauma. Secondary ABC may be associated with the progressive degeneration of pre-existing lesions such as a cyst, tumor, or fibro-osseous lesions such as solitary bone cyst, ossifying fibroma, and giant cell granuloma [4]. 

ABC is associated with some other type of bone lesion, which is presumed to be antecedent to it and considered as "ABC plus lesions." Various theories have explained the pathophysiology of ABC. The first theory suggests that a rise in vascular pressure and vasodilatation of the local vascular network due to regional circulatory dysregulation result in the formation of ABCs [1]. In another theory, ABC is considered a reactive process as most of the ABC cases occur with or inside the neoplasm. Neoplasms may be benign or malignant. This theory is most widely accepted [5]. According to several studies, ABC should be considered a secondary lesion since it may have obscured the underlying or pre-existing lesion due to its morphological changes [6]. 

Considering the nature of the other associated bony lesions, three stages are recognized in the formation of the ABC, First is the initial phase, with predominant osteolysis and non-characteristic appearance. Second is the growth phase, with the rapid growth of the tumor, marked bone destruction, and expansion of the bone. The tumor is not circumscribed, and bony septa are indistinct. Progressively, the first signs of a bony shell appear around the tumor and the last stage is the stabilization phase, with a well-defined unilocular or multilocular radiolucency with histological features of blood-filled sinusoidal spaces along with fibrocellular connective tissue stroma. 

Some ABCs ossify spontaneously and change into an osseous mass with an irregular trabecular pattern [7]. There are several theories regarding the pathogenesis of ABCs. In the last decade, these theories are challenged by Panoutsakopoulos et al. [8] and Dal Cin et al. [9], who observed that in 70% of ABCs, there is a recurrent cytogenic abnormality with a chromosomal translocation t (16; 17) (q22; p13). Oliveira et al. identified a genetic rearrangement in spindle cells that are present in the fibrous walls of an ABC and considered them neoplastic cells [10]. The genetic rearrangement is present in the spindle cells and not in the giant cells, osteoblasts, endothelial cells, and inflammatory cells. Numerous oncogenetic research are going on the neoplastic transformation of bone tumors to ABC, which showed that there is a constant specific cytopathogenic mechanism. Primary ABC can be described as a "clonal neoplastic disorder," with USP6 being its primary oncogene and spindle cells considered the neoplastic cells [10]. According to various oncogenetic studies, "primary" ABCs are neoplasms and not pseudocysts, whereas "secondary" ABCs are the actual pseudocysts with an absence of an oncogenetic association with primary ABCs. These are the result of a subsequent aneurysmal alteration (only seen histologically) brought on by trauma-induced bleeding and/or a reactive process in a separate original tumor [11].

This article represents the case of a mandibular secondary ABC plus lesion of significant size and also successfully reviewed 32 ABC plus cases since 1963. Management of our case is a conservative surgical approach aimed at limiting the esthetic and functional side effects in a young patient.

## Case presentation

**Figure 1:**
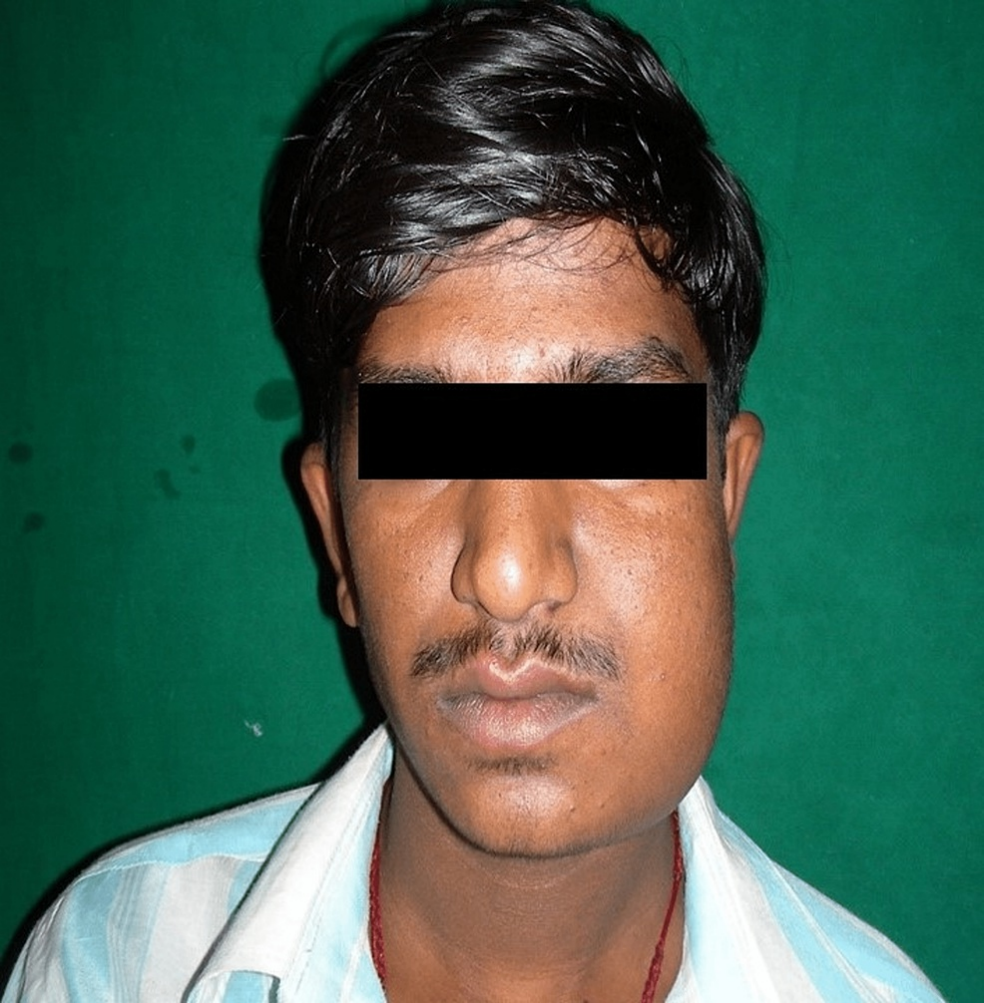
On external examination, there is facial asymmetry with localized single swelling measuring 10 x 8 cm on the left side

A 17-year-old male patient reported in our outpatient department (OPD) with the chief complaint of painless progressive swelling in the lower left posterior region of the jaw since three years. There was no significant medical history. Localized unifocal swelling gradually increased in size; previously, it was peanut sized, and it gradually increased up to 10 x 8 cm measuring approximately on the left side (Figure 1). 

**Figure 2:**
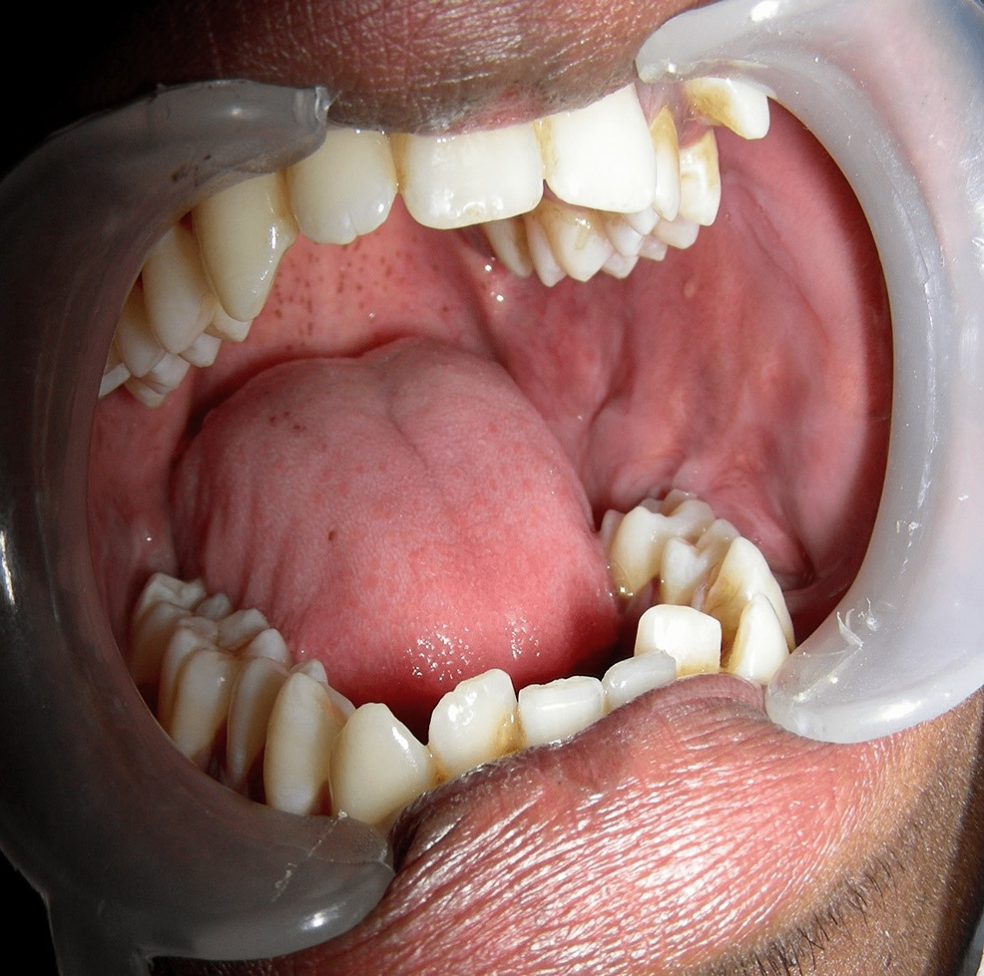
Intraoral examination shows expansion of buccal cortical plates and obliteration of buccal vestibule

The overlying skin of the lesion was normal; the intraorally buccal vestibule was obliterated. The swelling was bony hard and non-tender, extending from the lower left first premolar to the anterior border of the ramus. There was buccal cortical plate expansion. The lower left second and third molars were missing (Figure 2). 

**Figure 3:**
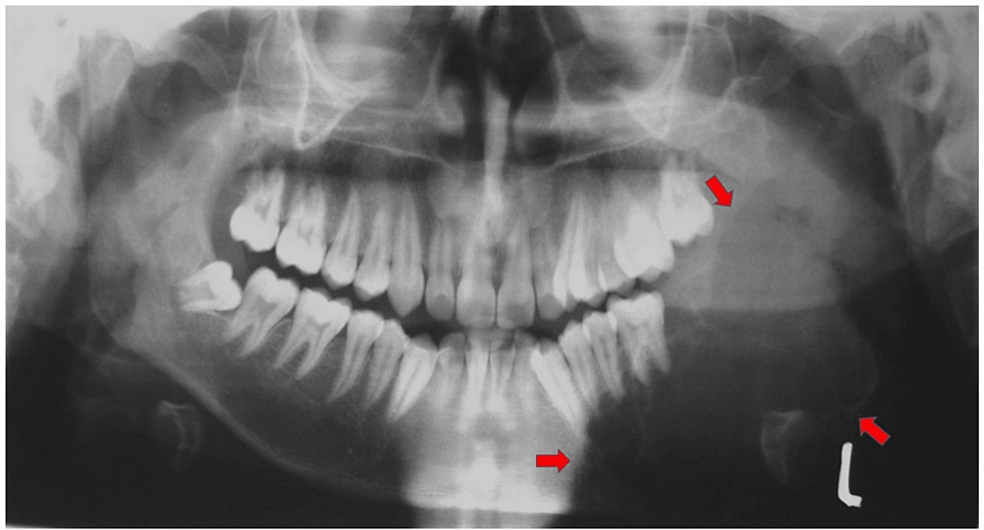
OPG shows a well-defined multilocular radiolucent, cystic lesion in the body and the ramus of the mandible on the left side. OPG, orthopantomogram.

On aspiration, less than 3 mL of serosanguineous fluid was obtained. The protein content was more than 5 g/dL. The orthopantomogram (OPG) showed well-defined unilocular radiolucency extending anteroposteriorly from the lower left canine to ramus and super inferiorly from sub condylar to the inferior border of the mandible (Figure 3). CT scan revealed a cystic lesion in the body and the ramus of the mandible on the left side.

**Figure 4:**
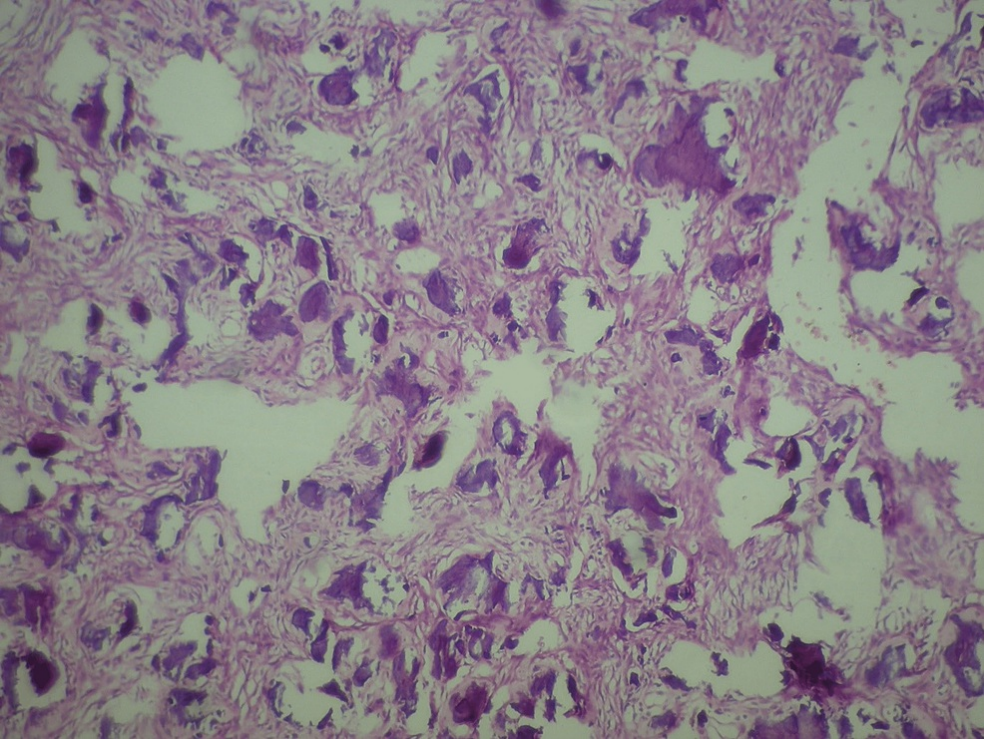
Histopathology shows fibrocellular stroma of variable grades of maturity in which numerous woven-type bony trabeculae were spread uniformly Hematoxylin and eosin-stained section at 100× magnification.

The histopathological pictures show fibrocellular stroma of variable grades of maturity in which numerous woven-type bony trabeculae were spread uniformly (Figure 4).

**Figure 5:**
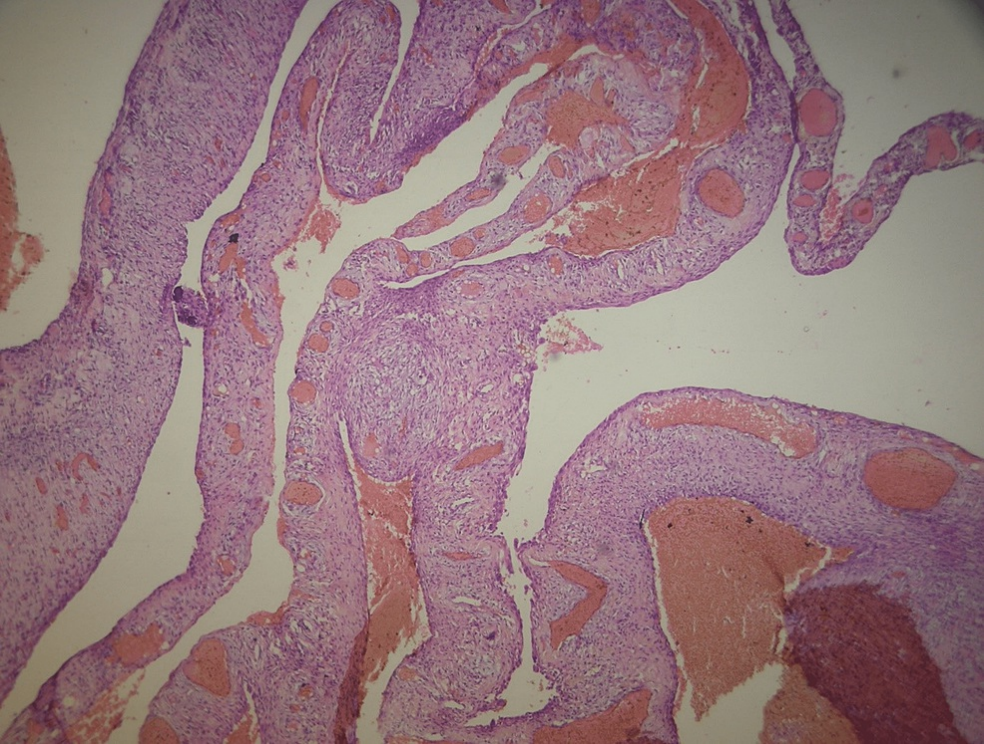
Histopathology shows fibrocellular connective tissue stroma consisting of numerous sinusoidal blood-filled spaces Hematoxylin and eosin-stained section at 100× magnification.

The trabeculae did not show the osteoblastic lining. The fibrocellular connective tissue stroma was consisting of numerous sinusoidal blood-filled spaces (Figure 5).

This is again a high-power view showing cementum-like material in the fibrocellular stroma, so the overall histopathological picture was suggestive of an ABC associated with cemento-ossifying fibroma. The postoperative OPG of the same patient after three months showed regeneration of bone. The patient was stable after surgery.

## Discussion

The term aneurysmatic refers to the blow-out effect or expansion of the affected bone in these lesions. The ABC of the jaw is described as a pseudocyst that lacks the epithelial lining. It represents 5% of all the lesions associated with the cranial and maxillofacial bones. It occurs most commonly in the skeleton region where there is comparatively more venous and marrow content. Skull bones have low venous pressure, making ABCs rare lesions in skull bones [12].

A review of the literature (Table 1) identified 32 ABC plus lesions; out of 32 cases, it was observed that it has a male predominance, and the mandible is more prone to being affected. More than 90% of ABC plus lesions are present in the posterior region of the jaw. Of 32 ABC plus lesions, 68% were associated with fibro-osseous lesions. Thirty-two percent of lesions were associated with giant cell lesions. ABC shows a varied range of clinical features, from an asymptomatic lesion, detected on the routine radiograph to a painful, expansive, and destructive pattern [13]. But most of the lesions were asymptomatic swelling which was also present in our case. It also shows male predominance, and the mandible and the posterior region of the jaw were affected.

**Table 1:** Review of ABC plus case reports by various authors ABC, aneurysmal bone cyst.

Sr. No	Author	Year/Sex	Site	Diagnosis
	Yarington et al. 1964 [14]	48/F	Maxilla	Giant cell (reparative granuloma)
	Costas and Pietropinto 1970 [15]	22/F	Mandible	Giant cell granuloma
	Buraczewski and Dabska 1971 [16]	26/F	Mandible	Fibrous dysplasia
	Ellis and Walter 1972 [17]	17/M	Maxilla	Cementifying fibroma
	Oliver 1973 [18]	20/F	Mandible	Fibrous dysplasia
	Bertrand et al. 1978 [19]	28/M	Mandible	Fibrous dysplasia ; Ossifying fibroma
	El Deeb et al. 1980 [20]	19/M	Mandible	Fibrous dysplasia
	Goldmann and Sisson 1980 [21]	10/M	Maxilla	Fibrous dysplasia
	Pankey et al. 1984 [22]	20/M	Mandible	Central giant cell granuloma
	Robinson et al. 1985 [23]	13/M	Mandible	Cementifying fibroma
	Sun et al. 2010 [24]	11/M	Maxilla	Cemento-ossifying fibroma
		41/M	Mandible	Cemento-ossifying fibroma
		11/M	Mandible	Cemento-ossifying fibroma
		14/F	Mandible	Cemento-ossifying fibroma
		18/F	Mandible	Benign osteoblastoma
		47/F	Mandible	Cemento-ossifying fibroma
		12/F	Maxilla	Cemento-ossifying fibroma
		17/M	Mandible	Ossifying fibroma
		27/F	Mandible	Ossifying fibroma
		30/F	Mandible	Ossifying fibroma
		9/M	Mandible	Central giant cell granuloma
		14/M	Mandible	Cemento-ossifying fibroma
		7/M	Mandible	Cemento-ossifying fibroma
	Sankaranarayanan et al. 2011 [25]	6/F	Maxilla	Juvenile ossifying fibroma
	Westbury et al. 2011 [26]	17/F	Maxilla	Central giant cell granuloma
	Tabrizi et al. 2011 [27]	26/M	Mandible	Non-ossifying fibroma
	Henriques et al. 2012 [28]	21/M; 18/M	Mandible; mandible	Ossifying fibroma; giant cell lesion
	Arora et al. [11]	61/M	Mandible	Giant cell granuloma
	Moghe et al. 2014 [12]	8/F	Maxilla	Ossifying fibroma
	Sachin C. Sarode 2018 [29]	10/M	Maxilla	Juvenile ossifying fibroma

In contrast to the ABC that occurred in long bones, ABC plus lesions frequently present with pain combined with rapid growth tendency, while ABCs that appear in the other parts of the body are commonly associated with malignant tumors such as chondrosarcomas and osteosarcomas [30]. Generally, ABCs occur in the jaw bones. They have never seen any malignant or metastatic lesions of the bone. They are also quite rarely associated with aggressive neoplasms such as osteoblastoma and benign ameloblastoma [19].

The radiological appearance of jaw ABC is quite inconsistent. The lesion may show bony expansion, a cystic appearance resembling a honeycomb or soap bubble, or be eccentrically ballooned. There may be perforation or destruction of the cortex, and a periosteal reaction may be manifested [31]. It may be a mixed, radiopaque, or radiolucent lesion. In the present case, we observed a unilocular radiolucency, which led to the expansion of the cortical plates and also caused thinning of the lower border of the mandible. Resorption of the roots of the involved teeth was also noticed. The diagnosis of the lesion by radiographical appearance is unfeasible since there are other lesions having a similar radiographic appearance, such as odontogenic cysts, ameloblastoma, central giant cell granuloma, and myxoma, or central hemangiomas of the bone [32]. Histopathologically, ABC consists of a large number of blood-filled sinusoidal spaces present in a fibrocellular connective tissue stroma, with osteoid material and multinucleated giant cells. Hemosiderin pigments are also present in variable amounts. This description is pathognomonic of the classic or vascular form of ABC [33]. In contrast, the solid form shows hemorrhagic foci with abundant fibroblastic and fibrohistiocytic elements and osteoclast-like giant cells, osteoblastic differentiation areas with osteoid, and calcifying fibromyxoid tissue. Both solid and vascular features are seen in the mixed form. The ABC plus lesions show a mixture of vascular as well as solid form, along with associated lesions; it shows numerous blood-filled spaces in fibrocellular stroma along with multinucleated giant cell and osteoid tissue formation [34]. In this case, histopathological features of the lesion were in accordance with the features mentioned above, which suggests an ABC with cemento-ossifying fibroma. 

Generally, the treatment plan of ABC plus lesion involves complete removal of the lesion. The lesions are usually multilocular and may divide by multiple bony septae. This makes it difficult for surgical removal. The treatment modalities are diagnostic and therapeutic embolization, curettage, block resection, radiotherapy reconstruction, and systemic calcitonin therapy. Some studies have reported a few self-healing cases after a long-term follow-up. In cases of esthetic deformity, loss of continuity of the mandible, and cases with a high risk of fractures, many authors have indicated immediate reconstruction of the defect using autogenous grafts for treatment. In the present case, the treatment was done by curettage and regular monitoring was also done. After one year of follow-up, no evidence of any residual lesion was seen.

## Conclusions

Frequently ABC may occur in association with fibro-osseous lesions which are categorized as ABC plus lesions. The present case of secondary ABC plus lesion was in context to clinical, radiographic, and histopathological parameters. Diagnosis and identification of ABC plus lesions and their further management are challenging for a surgeon because of its diverse pathogenesis. In this case, an incisional biopsy was done, but due to scanty tissue, the diagnosis was inconclusive. So, multiple sites of biopsies may be helpful for the initial diagnosis of the lesion so that appropriate treatment modalities can be instituted.

## References

[1] Jaffe HL, Lichtenstein L (1942). Solitary unicameral bone cyst with emphasis on the Roentgen picture, the pathologic appearance and pathogenesis. Arch Surg.

[2] Rosenberg AE, Nielsen GP, Fletcher JA (2002). World Health Organisation Classification of Tumors. Pathology and Genetics of Tumours of Soft tissues and Bone. Pathology and Genetics of Tumours of Soft tissues and Bone; p.

[3] Kalantar Motamedi MH (1998). Aneurysmal bone cyst of the jaws: clinicopathological features, radiographic evaluation and treatment analysis of 17 cases. J Craniomaxillofac Surg.

[4] Svensson B, Isacsson G (1993). Benign osteoblastoma associated with an aneurysmal bone cyst of the mandibular ramus and condyle. Oral Surg Oral Med Oral Pathol.

[5] Biesecker JL, Marcove RC, Huvos AG, Miké V (1970). Aneurysmal bone cyst: a clinic-pathologic study of 66 cases. Cancer.

[6] Kransdorf MJ, Sweet DE (1995). Aneurysmal bone cyst: concept, controversy, clinical presentation, and imaging. AJR Am J Roentgenol.

[7] Buraczewski J, Dabska M (1971). Pathogenesis of aneurysmal bone cyst, relationship between the aneurysmal bone cyst and fibrous dysplasia of bone. Cancer.

[8] Panoutsakopoulos G, Pandis N, Kyriazoglon I, Gustafson P, Mertens F, Mandahl N (1999). Recurrent t (16;17) (q22;p13) in aneurysmal bone cysts. Genes Chromosomes Cancer.

[9] Dal Cin P, Kozakewich HP, Goumnerova L, Mankin HJ, Rosenberg AE, Fletcher JA (2000). Variant translocations involving 16q22 and 17p13 in solid variant and extraosseous forms of aneurysmal bone cyst. Genes Chromosomes Cancer.

[10] Oliveira AM, Perez-Atayde AR, Inwards CY (2004). USP6 and CDH11 oncogenes identify the neoplastic cell in primary aneurysmal bone cysts and are absent in so-called secondary aneurysmal bone cysts. Am J Pathol.

[11] Arora SS, Paul S, Arora S, Kapoor V (2014 ). Secondary jaw aneurysmal bone cyst (JABC)--a possible misnomer? A review of literature on secondary JABCs, their pathogenesis and oncogenesis. J Oral Pathol Med.

[12] Moghe S, Saini N, Pillai A, Moghe A, Pillai K (2014). Aneurysmal bone cyst plus in an 8-year-old female, a case report. IOSR J Dent Med Sci.

[13] Kiattavorncharoen S, Joos U, Brinkschmidt C, Werkmeister R (2003). Aneurysmal bone cyst of the mandible: a case report. Int J Oral Maxillofac Surg.

[14] Yarington CT, Abbott J, Raines D (1964). Aneurysmal bone cyst of the maxilla; Association with giant cell reparative granuloma. Arch Otolaryngol.

[15] Costas JB, Pietropinto J (1970). [Aneuryismal bone cyst of the mandible]. An Esp Odontoestomatol.

[16] Buraczewski J, Dabska M (1971). Pathogenesis of aneurysmal bone cyst. Relationship between the aneurysmal bone cyst and fibrous dysplasia of bone. Cancer.

[17] Ellis DJ, Walters PJ (1972). Aneurysmal bone cyst of the maxilla. Oral Surg Oral Med Oral Patho.

[18] Oliver LP (1973). Aneurysmal bone cyst. Report of a case. Oral Surg Oral Med Oral Pathol.

[19] Bertrand G, Minard MF, Simard C, Rebel A (1978). [Ultrastructural study of a case of monostotic fibrous dysplasia]. Ann Anat Pathol (Paris).

[20] El Deeb M, Sedano HO, Waite DE (1980). Aneurysmal bone cyst of the jaws. Report of a case associated with fibrous dysplasia and review of the literature. Int J Oral Surg.

[21] Goldman ME, Sisson GA (1980). Fibrous dysplasia of maxilla with its relation to aneurysmal bone cyst. Proc Inst Med Chic.

[22] Pankey ER, Schaberg SJ, Pierce GL, Williams TP (1984). Clinicopathologic conference. Case 48, part II: Aneurysmal bone cyst of the mandible. J Oral Maxillofac Surg.

[23] Robinson PD (1985). Aneurysmal bone cyst: a hybrid lesion?. Br J Oral Maxillofac Surg.

[24] Sun ZJ, Zhao YF, Yang RL, Zwahlen RA (2010). Aneurysmal bone cysts of the jaws: analysis of 17 cases. J Oral Maxillofac Surg.

[25] Sankaranarayanan S, Srinivas S, Sivakumar P, Sudhakar R, Elangovan S (2011). "Hybrid" lesion of the maxilla. J Oral Maxillofac Pathol.

[26] Westbury SK, Eley KA, Athanasou N, Anand R, Watt-Smith SR (2011). Giant cell granuloma with aneurysmal bone cyst change within the mandible during pregnancy: a management dilemma. J Oral Maxillofac Surg.

[27] Tabrizi R, Nejhad ST, Özkan BT (2011). Nonossifying fibroma secondary to aneurysmal bone cyst in the mandibular condyle. J Craniofac Surg.

[28] Henriques AC, Carvalho Mde V, Miguel MC, Queiroz LM, Da Silveira EJ (2012). Clinical pathological analysis of nine cases of aneurysmal bone cyst of the jaws in a Brazilian population. Eur Arch Otorhinolaryngol.

[29] Sarode SC, Sarode GS, Ingale Y, Ingale M, Majumdar B, Patil N, Patil S (2018). Recurrent juvenile psammomatoid ossifying fibroma with secondary aneurysmal bone cyst of the maxilla: a case report and review of literature. Clin Pract.

[30] Hall EH, Naylor GD, Mohr RW, Warnock GR (1987). Early aggressive cemento-ossifying fibroma: a diagnostic and treatment dilemma. Oral Surg Oral Med Oral Pathol.

[31] Kaffe I, Naor H, Calderon S, Buchner A (1999). Radiological and clinical features of aneurysmal bone cyst of the jaws. Dentomaxillofac Radiol.

[32] Karabouta I, Tsodoulos S, Trigonidis G (1991). Extensive aneurysmal bone cyst of the mandible: surgical resection and immediate reconstruction: a case report. Oral Surg Oral Med Oral Pathol.

[33] Capote-Moreno A, Acero J, García-Recuero I, Ruiz J, Serrano R, de Paz V (2009). Giant aneurysmal bone cyst of the mandible with unusual presentation. Med Oral Patol Oral Cir Bucal.

[34] Devi P, Thimmarasa V, Mehrotra V, Agarwal M (2011). Aneurysmal bone cyst of the mandible: a case report and review of literature. J Oral Maxillofac Pathol.

